# Case report: further delineation of *AEBP1*-related Ehlers–Danlos Syndrome (classical-like EDS type 2) in an additional patient and comprehensive clinical and molecular review of the literature

**DOI:** 10.3389/fgene.2023.1102101

**Published:** 2023-05-05

**Authors:** Tomomi Yamaguchi, Shujiro Hayashi, So Nagai, Akihiko Uchiyama, Sei-Ichiro Motegi, Tomomi Fujikawa, Yuri Takiguchi, Tomoki Kosho

**Affiliations:** ^1^ Center for Medical Genetics, Shinshu University Hospital, Matsumoto, Japan; ^2^ Department of Medical Genetics, Shinshu University School of Medicine, Matsumoto, Japan; ^3^ Division of Clinical Sequencing, Shinshu University School of Medicine, Matsumoto, Japan; ^4^ Department of Dermatology, Dokkyo Medical University, Mibu, Japan; ^5^ Problem-Solving Oriented Training Program for Advanced Medical Personnel: NGSD (Next-Generation Super Doctor) Project, Matsumoto, Japan; ^6^ Department of Dermatology, Gunma University Graduate School of Medicine, Maebashi, Japan; ^7^ Research Center for Supports to Advanced Science, Shinshu University, Matsumoto, Japan

**Keywords:** Ehlers-Danlos Syndrome, classical-like EDS type 2 (clEDS2), adipocyte enhancer binding protein 1 (*AEBP1*), aortic carboxypeptidase-like protein (ACLP), autosomal recessive, connective tissue disorders

## Abstract

The Ehlers–Danlos Syndromes (EDS), a group of hereditary connective tissue disorders, were classified into 13 subtypes in the 2017 International Classification. Recently, a new subtype of EDS called classical-like EDS type 2 (clEDS2), which is caused by biallelic variants in the adipocyte enhancer binding protein 1 (*AEBP1*) gene, was identified. We describe the 11th patient (9th family) with clEDS2, who was complicated by a critical vascular event (superior mesenteric artery aneurysm and rupture). A next-generation sequencing panel-based analysis revealed compound heterozygous variants in *AEBP1*: NM_001129.5:c.[2296G>T]; [2383dup], p.[(Glu766*)]; [(Glu795Glyfs*3)]. Light microscopic analyses showed increased interfibrillar spaces in the reticular dermis, a disorganized arrangement of collagen fibers, and decreased collagen content. An electron microscopic analysis showed the presence of collagen fibrils with irregular contours (flower-like appearance) and small collagen fibrils. A biochemical analysis showed reduced secretion of type I and type III procollagen. Clinical and molecular features of the current patient and all previously reported patients were reviewed comprehensively. Manifestations noted in most cases (>80%) included skin features (hyperextensibility, atrophic scars, easy bruising, excessive skin/skin folding, delayed wound healing, translucency, piezogenic papules), skeletal features (generalized joint hypermobility, dislocations/subluxations, pes planus), dental abnormalities, and neuromuscular abnormalities. Critical complications, each occurring in a single case, included superior mesenteric artery multiple aneurysm and rupture, aortic root dilation requiring surgery, and bowel rupture. Most *AEBP1* variants were predicted or experimentally confirmed to lead to nonsense-mediated mRNA decay, whereas one variant resulted in a protein that was retained intracellularly and not secreted. Clinical, molecular, pathological, and biochemical features of the current patient, as well as a review of all previously reported patients, suggest the importance of the aortic carboxypeptidase-like protein encoded by *AEBP1* in collagen fibrillogenesis.

## Introduction

The Ehlers–Danlos Syndromes (EDS) are a group of hereditary connective tissue disorders (HCTDs) characterized by skin hyperextensibility, joint hypermobility, and tissue fragility. They were classified into 13 subtypes based on symptoms and causative genes in the 2017 International Classification (Malfait et al., 2017). In 2018, Blackburn et al. (2018) identified biallelic variants in the adipocyte enhancer binding protein 1 (*AEBP1*) gene in patients displaying EDS-like features that were considered to represent a new subtype of EDS and were tentatively named classical-like type 2 (clEDS2; MIM #618000) (Malfait et al., 2020). To date, 10 patients from eight families have been described (Alazami et al., 2016; Blackburn et al., 2018; Hebebrand et al., 2019; Ritelli et al., 2019; Syx et al., 2019; Maddirevula et al., 2020; Vishwanath et al., 2020; Di Giosaffatte et al., 2022).

We report here an additional patient with clEDS2 who had novel variants in *AEBP1* and was complicated by a critical vascular event.

## Case description and molecular, pathological, and biochemical analysis

**FIGURE 1:**
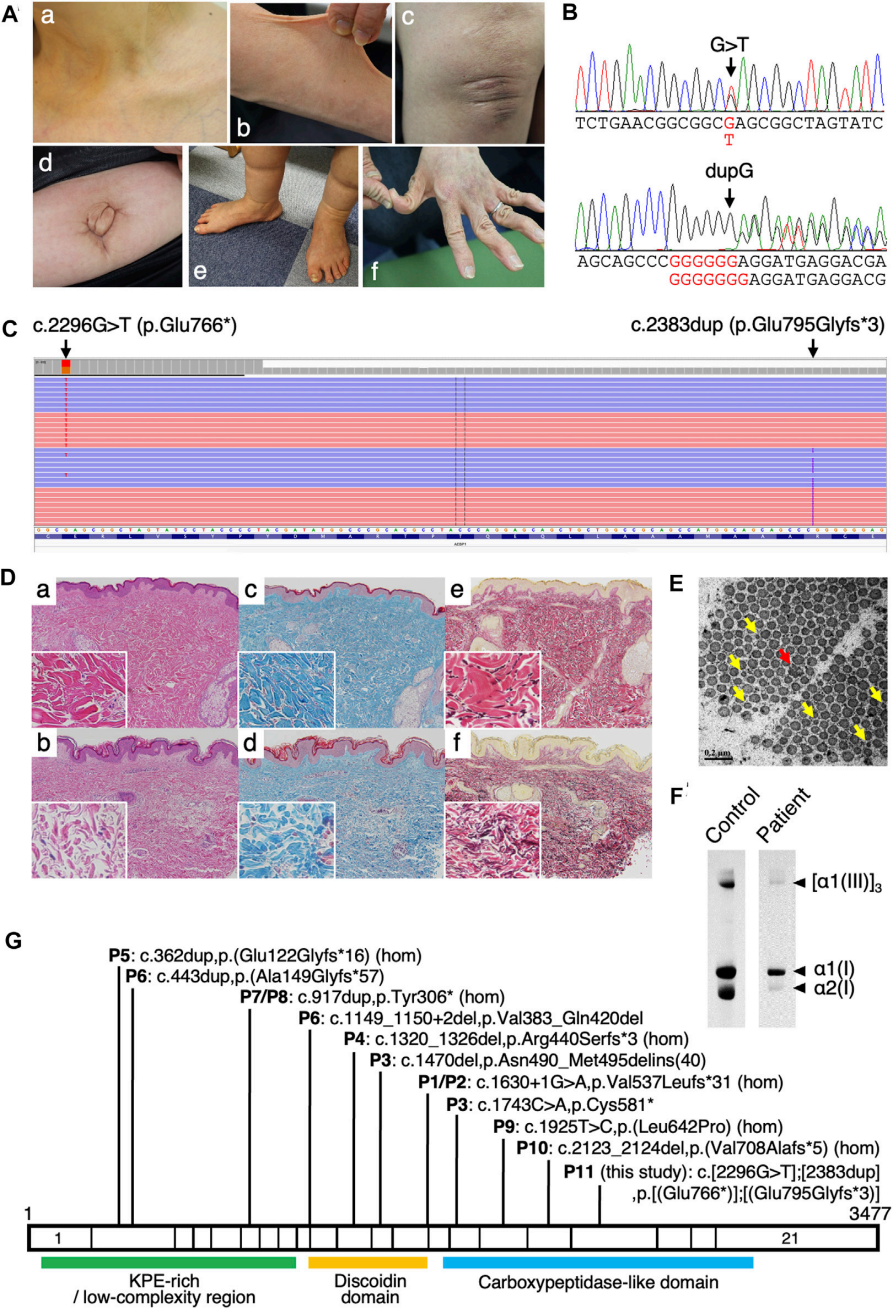
Clinical, molecular, histological, ultrastructural, and biochemical findings of the 11th patient identified with classical-like Ehlers–Danlos syndrome type 2 (clEDS2). **(A)** Clinical photographs of the current patient (Patient 11). Translucent skin on the upper chest at the age of 36 years (a). At age 45, the patient exhibited hyperextensible skin (b); a small atrophic scar (black arrow) and mild keloid formation (arrowhead) (c); an umbilical hernia (d); pes planus and edematous lower legs (e); and acrogeria-like skin on the hands, with hypermobile phalangeal joints (f). **(B)** Sanger sequencing electropherogram of variants in the adipocyte enhancer binding protein 1 gene (*AEBP1*). **(C)** Integrative Genomics Viewer visualization of the variants. The nonsense variant c.2296G>T and the frameshift variant c.2383dup are observed *in trans*. **(D)** Light microscopic images of a biopsy skin specimen (age 36) at two magnifications: low- (×20) and higher-power (×400, inset) magnifications. Hematoxylin and eosin staining showing increased spacing and disorganization of collagen fibers (b) compared with an age- and sex-matched control (a), Masson’s trichrome staining showing decreased collagen fibers (blue) (d) compared with control (c), and Elastica van Gieson staining showing collagen fibers (red) and elastic fibers (black) (f) compared with control (e). **(E)** Transmission electron microscopic images of the dermal collagen fibrils, showing irregular contours (flower-like appearance, red arrows) and small-sized fibrils (yellow arrows). **(F)** Type I and type III procollagen produced by cultured skin fibroblasts obtained from the patient and an age- and sex-matched control. **(G)** Schematic representation of the distribution of *AEBP1* variants in 11 patients from nine families with clEDS2 illustrated on *AEBP1* mRNA; “1” and “3477” indicate the first and the last nucleotide positions of the coding region of the mRNA, respectively. Domains of the aortic carboxypeptidase-like protein (ACLP) are shown at the corresponding exons. hom: homozygote; P1–P11: Patients 1–11; KPE-rich: lysine, proline and glutamic acid-rich.

The patient, a 45-year-old Japanese woman, was the second child of non-consanguineous parents. No skin hyperextensibility, fragility, or joint hypermobility were noted in her mother, elder sister, or two daughters. She was a preterm and low-birth-weight (1,980 g) infant. The patient had bilateral congenital hip dislocation for which she underwent fixation with a brace, experienced repetitive episodes of skin lacerations and subcutaneous hemorrhage after minor trauma, and had marked joint laxity, with repetitive sprains caused by unstable ankle joints. In her early 20 s, she was suspected to have EDS. Intractable hair loss has been her major physical concern since around that age. She also had spinal disc herniation. At the age of 36 years, she developed massive intraabdominal hemorrhages caused by rupture of the superior mesenteric artery, which were associated with multiple aneurysms and were treated with catheter embolization. Thin translucent skin was noted (Figure 1A-a).

When the patient was referred to us at the age of 45 years, she exhibited the following characteristics: hair with a kinky texture and generalized thinning; a high palate and multiple dental caries; hyperextensible and translucent skin (Figure 1A-a, b); skin striae in the lower extremities, with atrophic scars (Figure 1A-c); soft soles; an umbilical hernia (Figure 1A-d); pes planus (Figure 1A-e); and generalized joint hypermobility (Beighton score 8/9) (Figure 1A-f). Radiological examination showed no spinal deformities. There had been no episodes of dislocations or musculoskeletal pain. No aortic root dilatation or valve abnormalities were detected on echocardiography. She had high myopia, but no hearing impairment.

Genomic DNA was extracted from peripheral blood using a QIAamp DNA Blood Mini Kit on a QIAcube (Qiagen, Valencia, CA, United States). A next-generation sequencing (NGS) panel-based analysis was performed on an Ion Torrent system (Ion Chef and Ion GeneStudio S5, Thermo Fisher Scientific, Waltham, MA, United States) using an Ion AmpliSeq custom panel for 52 genes associated with EDS and other HCTDs (Supplementary Table S1). Detected variants were annotated by SnpEff and SnpSift (https://snpeff.sourceforge.net/) using the processed vcf file of the Genome Aggregation Database (gnomAD) v2.1.1 (https://gnomad.broadinstitute.org/downloads), ToMMo 8.3KJPN Genotype Frequency Panel (v20200831) (https://jmorp.megabank.tohoku.ac.jp/202008/downloads#variant) (Tadaka et al., 2019), ClinVar (ftp://ftp.ncbi.nlm.nih.gov/pub/clinvar/vcf_GRCh37/clinvar_20220328), dbNSFP3.4c and dbscSNV1.1 (https://sites.google.com/site/jpopgen/dbNSFP). Detected variants were evaluated in accordance with the 2015 American College of Medical Genetics and Genomics/Association for Molecular Pathology (ACMG/AMP) guidelines (Richards et al., 2015) and the ClinGen Sequence Variant Interpretation Working Group recommendations (SVI recommendations). Integrative Genomics Viewer (IGV) was used to visualize read alignments (Broad Institute, Cambridge, MA, United States). The NGS panel-based analysis revealed a non-sense variant c.2296G>T,p.(Glu766*) and a frameshift variant c.2383dup,p.(Glu795Glyfs*3) in *AEBP1* (NM_001129.5), which were confirmed by Sanger sequencing (Figure 1B). The IGV revealed that the two variants were observed *in trans* (Figure 1C). The nonsense variant was registered in 8.3KJPN (1/16758, MAF = 0.0001, no homozygote) and the frameshift variant was registered in 8.3KJPN (1/16754, MAF = 0.0001, no homozygote). Both variants were classified as pathogenic (PVS1, PM2_Supporting, and PM3), in accordance with the 2015 ACMG/AMP guidelines and SVI recommendations.

Hematoxylin and eosin staining of a skin specimen obtained by a biopsy performed at the age of 36 years showed increased spaces between collagen fibers and disorganized orientations of these fibers in the lower and middle layer of the dermis (Figure 1D-b) compared with control (Figure 1D-a). Masson’s trichrome staining revealed decreased collagen fibers (Figure 1D-d) compared with control (Figure 1D-c). Elastica van Gieson staining showed prominent elastic fibers due to the decreased numbers of collagen fibers (Figure 1D-f) compared with control (Figure 1D-e). An ultrastructural analysis using transmission electron microscopy of the skin specimen revealed the presence of collagen fibrils with irregular contours (flower-like appearance) and small size under the cross-sectional view (Figure 1E). Measurement of procollagen production from cultured skin fibroblasts was performed as described previously (Shimaoka et al., 2010). Briefly, dermal fibroblasts from the patient were incubated with ^3^H-proline for 24 h. Labeled proteins secreted into the culture medium were digested with pepsin and analyzed by sodium dodecyl sulfate-polyacrylamide gel electrophoresis/fluorography. Amounts of type I and type III procollagen were both reduced compared with an age- and sex-matched individual who served as a control (Figure 1F).

## Discussion

We have identified and described a 11th patient (9th family) with clEDS2, who was found to have novel compound heterozygous pathogenic variants in *AEBP1*. Detailed and comprehensive clinical and molecular features of all previously reported patients and the current patient are shown in Table 1.

**TABLE 1:** Detailed and comprehensive clinical and molecular features of all previously reported patients and the current patient.

Family no.	I		II	III	IV	V	VI		VII	VIII	IX	
Patient No.	1	2	3	4	5	6	7	8	9	10	11	
Citation(s)	Alazami et al. (2016)		Blackburn et al. (2018)		Syx et al. (2019)		Hebebrand et al. (2019)		Ritelli et al. (2019)	Di Giosaffatte et al. (2022)	This report	
	Maddirevula et al. (2020)		Vishwanath et al. (2020)									
Age at the time of report (years)	12	24	35	33	58	21	39	38	53	26	45	
Sex	Female	Male	Male	Male	Male	Female	Female	Male	Female	Male	Female	
Ethnicity	Middle Eastern	Middle Eastern	Caucasian	Caucasian	Caucasian	Caucasian	Greek	Greek	Italian	NA	Japanese	
*AEBP1* variant (NM_001129.5)	c.[1630 + 1G>A];[1630 + 1G>A]	c.[1630 + 1G>A];[1630 + 1G>A]	c.[1470del];[1743C>A]	c.[1320_1326del];[1320_1326del]	c.[362dup];[362dup]	c.[443dup];[1149_1150+2del]	c.[917dup];[917dup]	c.[917dup];[917dup]	c.[1925T>C];[1925T>C]	c.[2123_2124del];[2123_2124del]	c.[2296G>T];[2383dup]	
Protein alteration (NP_001120.3)	p.[Val537Leufs*31];[Val537Leufs*31]	p.[Val537Leufs*31];[Val537Leufs*31]	p.[Asn490_Met495delinsLysAlaMetArgLysTrpTrpAlaProCysProGlySerTrpLeuCysSerHisCysLeuGlyGluGlyTrpAlaLeuArgGlyAlaGlySerThrAlaLeuArgProAlaSerProGln];[Cys581*]	p.[Arg440Serfs*3];[Arg440Serfs*3]	p.[(Glu122Glyfs*16)];[(Glu122Glyfs*16)]	p.[(Ala149Glyfs*57)];[Val383_Gln420del]	p.[Tyr306*];[Tyr306*]	p.[Tyr306*];[Tyr306*]	p.[(Leu642Pro)];[(Leu642Pro)]	p.[(Val708Alafs*5)];[(Val708Alafs*5)]	p.[(Glu766*)];[(Glu795Glyfs*3)]	
Craniofacial features	Bilateral ptosis, webbed neck, low posterior hairline, sagging cheeks, large ears, narrow/high palate	Bilateral ptosis, webbed neck, low posterior hairline, sagging cheeks, large ears, narrow palate	−	Micrognathia	Asymmetrical face, hypertelorism, low-set and posteriorly rotated ears with attached earlobes, thin and frizzled hair, partial alopecia, webbed neck	Mild ptosis, thinning hair	Alopecia	NA	Alopecia, high palate, elongated uvula	Cleft palate, down-slanting palpebral fissures, epicanthus, deep set eyes, malar hypoplasia low set ears, micro/retrognathia, webbed neck	Thinning and kinky hair, high palate, narrow nose	Alopecia or thinning hair
												5/10 (50.0%)
Dental features	Abnormal dental alignment	Abnormal dental alignment	Retains a single baby tooth	NA	Bad tooth quality with severe caries	Bad tooth quality with frequent caries	NA	NA	Pyorrhea, complete dental loss at age 14	Multiple caries, periodontal disease	Multiple caries	8/8 (100%)
Cutaneous features												
Skin hyperextensibility	+	+	+	+	+	+	+	+	+	+	+	11/11 (100%)
Thin, translucent skin	NA	NA	NA	+	+	+	+	+	−	+	+	7/8 (87.5%)
Excessive skin	+	+	+	+	+	−	+	+	+	+	+	10/11 (90.9%)
/skin folding												
Delayed wound healing	+	+	+	+	+	Mild	+	+	+	−	+	10/11 (90.9%)
Atrophic scars	+	NA	+	+	+	+	+	+	+	+	+	10/10 (100%)
Easy bruising	+	NA	+	+	+	+	+	+	+	+	+	10/10 (100%)
Piezogenic papules	NA	NA	+	+	NA	NA	NA	NA	+	+	−	4/5 (80.0%)
Prematurely aged appearance	NA	NA	− (Increased acrogeria-like skin wrinkles on hands and feet)	NA	+	NA	+	+	+	−(Acrogeria-like hand appearance)	− (Acrogeria-like skin on hands)	4/7 (57.1%)
Other				Sacral dimple	Decubitus wounds on buttocks		Skin striae	Fragile skin lesions on the buttocks		Skin fragility, subcutaneous spheroids, palmar callosities	Skin lacerations after minor trauma, skin striae, velvety skin	
Skeletal features												
Generalized joint hypermobility (Beighton score)	+ (8/9)	+ (NA)	+ (8/9)	+ (8/9)	+ (NA)	+ (9/9)	+ (6/9)	− (2/9)	+ (5/9)	+ (7/9)	+ (8/9)	10/11 (90.9%)
Congenital hip dislocation	−	−	−	+	−	−	−	−	−	−	+	2/11 (18.2%)
Other dislocations	Interphalangeal/hip/knee/ankle	Hip/knee/ankle	Distal radioulnar joint	Shoulder	Elbow	−	Wrist	Clavicular/knee/ankle	Shoulder/elbow/knee/ankle	−	−	10/11 (90.9%)
Subluxations	−	−	Shoulder/hip	−	−	Temporomandibular/shoulder/elbow/thumb/hip/knee	Mandibular	−	−	+	−	
Pectus excavatum	−	NA	−	−	+	−	−	+	−	−	−	2/10 (20.0%)
Spine deformities	−	NA	−	Thoracic scoliosis with degenerative disease and facet arthrosis of spine	−	Scoliosis	Kyphoscoliosis	Kyphoscoliosis	Scoliosis (mild)	Scoliosis	−	6/10 (60.0%)
Pes planus	+	+	+	+	+	+	Mild	+	+	−	+	10/11 (90.9%)
Hallux valgus	+	+	+	+	+	−	−	−	+	−	−	6/11 (54.5%)
Hammertoes	+	+	+	+	+	−	−	−	−	+	−	6/11 (54.5%)
Osteopenia	+	+	+	+	NA	−	NA	NA	+	−	NA	5/7 (71.4%)
Other				Downsloping shoulders, severe degenerative disease requiring hip replacement	Multiple ankle distortions	Hip dysplasia, ankle sprains, drooping shoulders	Arachnodactyly, wrist and thumb signs, systemic score 8	Hindfoot deformity, arachnodactyly, wrist sign, systemic score 7	Patellar instability, gonarthrosis, rotator cuff disease, achilles tendinopathy, subacromial shoulder impingement, epitrochleitis	Short stubby fingers, hips dysmetria, absence and hypoplasia of toes	Toe/elbow joint deformity, ankle instability, sprains	
Neuromuscular features	Neonatal hypotonia	Myopathy	Delays in walking and acquisition of fine motor skills	NA	NA	Progressive decreased muscle strength, inability to walk without support	NA	NA	Neonatal severe hypotonia, delays in walking and acquisition of fine motor skills, hypotrophy of the scapular girdle	Mild perinatal hypotonia, delayed motor development, unilateral hypoplasia of right pectoralis major muscle, diastasis recti	NA	6/6 (100%)
Cardiovascular features	−	−	Mitral valve prolapse	Mild mitral regurgitation, bilateral stenosis of the carotids, aortic root dilation	Mild mitral valve prolapse	Vaginal hematoma after trauma, postural orthostatic tachycardia syndrome	Mitral valve prolapse, circular pericardial effusion	Varicose veins	Peripheral artery disease (intermittent claudication, peripheral cyanosis, cold skin), varicose veins	Mild regurgitation at tricuspid, pulmonary, and mitral valves, varicose veins, hematoma	Superior mesenteric artery aneurysm and rupture	
Gastrointestinal features	NA	NA	Chronic constipation	Bowel rupture	−	Gastroesophageal reflux, esophageal spasms, dysphagia, bloated feeling, abdominal cramps/pain and episodes of constipation or diarrhea	NA	NA	−	NA	−	
Hernias	Umbilical/ventral/inguinal hernia	NA	−	Large ventral hernia developed at surgical sites secondary to ruptured bowel	Herniation of fat in the right armpit	NA	+	−	Umbilical hernia	Inguinal hernia	Spinal disc herniation, umbilical hernia	7/9 (77.8%)
Urogenital features	NA	NA	Cryptorchidism	NA	NA	Urinary retention requiring catheterization, bladder cramps and urinary urgency	NA	Cryptorchidism	NA	Cryptorchidism	−	
Other features	Diabetes mellitus, cellulitis		Impaired temperature sensation, keratoconjunctivitis sicca	Elbow bursitis, hypertriglyceridemia	Spontaneous pneumothorax, myopia, tinnitus	Chronic fatigue, chronic widespread pain, mild myopia		Strabismus, astigmatism, myopia	Vocal cord nodules, subcutaneous spheroids, multiple papules with follicular prominence, chronic fatigue, myopia, astigmatism	Musculoskeletal back pain	Myopia	

*AEBP1*: adipocyte enhancer binding protein 1 gene; NA: not applicable; +: present; −: absent.

Dental abnormalities, skin hyperextensibility, atrophic scars, easy bruising, and neuromuscular abnormalities were observed in all patients whose data were available. Excessive skin/skin folding (90.9%), delayed wound healing (90.9%) generalized joint hypermobility (90.9%), dislocations/(sub)luxations (90.9%), pes planus (90.9%), translucent skin (87.5%), piezogenic papules (80.0%), hernia (77.8%), osteopenia (71.4%), spine deformities (60.0%), prematurely aged appearance (57.1%), hallux valgus (54.5%), and hammertoes (54.5%) were observed in more than half of the patients whose data were available. Decreased hair described as “thinning” or “(partial) alopecia” was observed in five patients, and was a major physical concern in the current patient. The current patient developed multiple aneurysms and a rupture in the superior mesenteric artery, which was treated with catheter embolization. Cardiovascular complications reported in the previous patients included mitral valve prolapse/regurgitation, tricuspid valve regurgitation, pulmonary valve regurgitation, varicose veins, and aortic root dilation requiring surgery. A bowel rupture occurred in one patient, requiring repeated attempts to re-anastomose the bowel and colostomy. This is the first report of skin lacerations, which occurred in the current patient, whereas skin fragility was only noted in two other patients. Heterozygous individuals appear to have no relevant symptoms.

Most reported *AEBP1* variants were null variants, including nonsense, frameshift and splice site variants, predicted to lead to nonsense-mediated mRNA decay (NMD) (Table 1; Figure 1G). Some variants were experimentally confirmed to affect the gene product. In Patients 1 and 2, a homozygous splice site variant (c.1630 + 1G>A) led to activation of the cryptic 5′splice site within exon 13 and skipping of the last 22 bp of exon 13. The shift in reading frame (p.Val537Leufs*31) was predicted to lead to NMD. In Patient 3, a 1-bp deletion (c.1470del) in exon 12 in one allele led to the retention of intron 12 (p.Asn490_Met495delinsLysAlaMetArgLysTrpTrpAlaProCysProGlySerTrpLeuCysSerHisCysLeuGlyGluGlyTrpAlaLeuArgGlyAlaGlySerThrAlaLeuArgProAlaSerProGln) (Blackburn et al., 2018). Its protein was retained intracellularly and not secreted (Vishwanath et al., 2020). A non-sense variant (c.1743C>A,p.Cys581*) in the other allele in Patient 3 was predicted to lead to NMD. In Patient 4, a homozygous frameshift variant (c.1320_1326del) led to a shift in reading frame (p.Arg440Serfs*3) (Blackburn et al., 2018). No ACLP protein was detected by western blotting, suggesting NMD. In Patient 6, a 4-bp deletion (c.1149_1150+2del) in one allele led to the loss of the last 4 bp of exon 9 and skipping of exon 10, resulting in an in-frame deletion (p.Val383_Gln420del) (Syx et al., 2019). A frameshift variant (c.443dup) in the other allele in Patient 6 was predicted to lead to NMD. The mRNA expression was significantly decreased, indicating that the *AEBP1* transcript was unstable and/or prone to NMD.

In the current patient, light microscopic analyses showed increased interfibrillar spaces in the reticular dermis, a disorganized arrangement of collagen fibers and decreased collagen content, and a biochemical analysis showed reduced secretion of type I and type III procollagen. Electron microscopic analysis showed the presence of collagen fibrils with irregular contours (flower-like appearance) and small size. Blackburn et al. (2018) reported that light microscopy showed decreased collagen, while electron microscopy revealed the presence of irregular disrupted collagen fibrils. In their report, the discoidin domain, a highly conserved structural motif of ACLP, preferentially bound to collagen types I, III and V, and ACLP promoted the polymerization of type I collagen *in vitro*. Syx et al. (2019) reported electron microscopic observations that corresponded with those of Blackburn et al. (2018) and the current study, and a biochemical analysis that showed a normal electrophoretic pattern of procollagen types I, III and V. This biochemical analysis was performed on the medium of cultured skin fibroblasts to detect procollagen secreted from these fibroblasts. The discrepant results might be attributable to some functional differences between the variants reported by Syx et al. and the variants in the current patient, which could be related to the difference in the transcription status of these procollagen genes or in the secretion status of these types of procollagen. In view of all of these findings, ACLP protein is likely an important player in collagen fibrillogenesis.

In conclusion, the clinical findings and disease course in the current patient, together with the review of previously reported patients, paints a picture of the clinical similarities and variations in clEDS2. Furthermore, the biochemical and pathological findings in the current patient, in addition to the relevant findings in the previous patients, suggest the importance of ACLP in collagen fibrillogenesis. Further clinical, molecular, and pathophysiological studies are required to produce a more detailed and comprehensive delineation of this disorder.

## Data Availability

The datasets for this article are not publicly available due to concerns regarding participant/patient anonymity. Requests to access the datasets should be directed to the corresponding authors.

## References

[B1] Alazami A. M. Al-Qattan S. M. Faqeih E. Alhashem A. Alshammari M. Alzahrani F. (2016). Expanding the clinical and genetic heterogeneity of hereditary disorders of connective tissue. Hum. Genet. 135, 525–540. 10.1007/s00439-016-1660-z 27023906

[B2] Blackburn P. R. Xu Z. Tumelty K. E. Zhao R. W. Monis W. J. Harris K. G. (2018). Bi-allelic alterations in *AEBP1* lead to defective collagen assembly and connective tissue structure resulting in a variant of Ehlers–Danlos syndrome. Am. J. Hum. Genet. 102, 696–705. 10.1016/j.ajhg.2018.02.018 29606302 PMC5985336

[B3] Di Giosaffatte N. Ferraris A. Gaudioso F. Lodato V. Savino E. Celletti C. (2022). Congenital defects in a patient carrying a novel Homozygous *AEBP1* Variant: Further expansion of the phenotypic spectrum of Ehlers-Danlos syndrome classical-like type 2? Genes (Basel) 13, 2358. 10.3390/genes13122358 36553625 PMC9777638

[B4] Hebebrand M. Vasileiou G. Krumbiegel M. Kraus C. Uebe S. Ekici A. B. (2019). A biallelic truncating *AEBP1* variant causes connective tissue disorder in two siblings. Am. J. Med. Genet. A 179, 50–56. 10.1002/ajmg.a.60679 30548383

[B5] Maddirevula S. Kuwahara H. Ewida N. Shamseldin H. E. Patel N. Alzahrani F. (2020). Analysis of transcript-deleterious variants in mendelian disorders: Implications for RNA-based diagnostics. Genome Biol. 21, 145. 10.1186/s13059-020-02053-9 32552793 PMC7298854

[B6] Malfait F. Castori M. Francomano C. A. Giunta C. Kosho T. Byers P. H. (2020). The ehlers–danlos syndromes. Nat. Rev. Dis. Prim. 6, 64. 10.1038/s41572-020-0194-9 32732924

[B7] Malfait F. Francomano C. Byers P. Belmont J. Berglund B. Black J. (2017). The 2017 international classification of the Ehlers–Danlos syndromes. Am. J. Med. Genet. C Semin. Med. Genet. 175, 8–26. 10.1002/ajmg.c.31552 28306229

[B8] Richards S. Aziz N. Bale S. Bick D. Das S. Gastier-Foster J. (2015). Standards and guidelines for the interpretation of sequence variants: A joint consensus recommendation of the American College of medical Genetics and Genomics and the association for molecular Pathology. Genet. Med. 17, 405–424. 10.1038/gim.2015.30 25741868 PMC4544753

[B9] Ritelli M. Cinquina V. Venturini M. Pezzaioli L. Formenti A. M. Maria A. (2019). Expanding the clinical and mutational spectrum of recessive *AEBP1*-related classical-like Ehlers–Danlos syndrome. Genes (Basel) 10, 135. 10.3390/genes10020135 30759870 PMC6410021

[B10] Shimaoka Y. Kosho T. Wataya-Kaneda M. Funakoshi M. Suzuki T. Hayashi S. (2010). Clinical and genetic features of 20 Japanese patients with vascular-type Ehlers–Danlos syndrome. Br. J. Dermatol. 163, 704–710. 10.1111/j.1365-2133.2010.09874.x 20518783

[B11] Syx D. De Wandele I. Symoens S. De Rycke R. Hougrand O. Voermans N. (2019). Bi-allelic *AEBP1* mutations in two patients with Ehlers–Danlos syndrome. Hum. Mol. Genet. 28, 1853–1864. 10.1093/hmg/ddz024 30668708

[B12] Tadaka S. Katsuoka F. Ueki M. Kojima K. Makino S. Saito S. (2019). 3.5KJPNv2, an allele frequency panel of 3,552 Japanese individuals including the X chromosome. Hum. Genome Var. 6, 28. 10.1038/s41439-019-0059-5 31240104 PMC6581902

[B13] Vishwanath N. Monis W. J. Hoffmann G. A. Ramachandran B. DiGiacomo V. Wong J. Y. (2020). Mechanisms of aortic carboxypeptidase-like protein secretion and identification of an intracellularly retained variant associated with Ehlers–Danlos syndrome. J. Biol. Chem. 295, 9725–9735. 10.1074/jbc.RA120.013902 32482891 PMC7363150

[B14] Yamaguchi T. Hayashi S. Hayashi D. Matsuyama T. Koitabashi N. Ogiwara K. (2022). Comprehensive genetic screening for vascular Ehlers–Danlos syndrome through an amplification-based next generation sequencing system. Am. J. Med. Genet. A. Online ahead print 191, 37–51. 10.1002/ajmg.a.62982 PMC10092364 36189931

