# Supplementary material for: Case report: further delineation of AEBP1-related Ehlers–Danlos Syndrome (classical-like EDS type 2) in an additional patient and comprehensive clinical and molecular review of the literature
Source: Front Genet. 2023 May 5;14:1102101. doi: 10.3389/fgene.2023.1102101 (PMC10196838; doi:10.3389/fgene.2023.1102101)
Supplement: Supplementary file 1 [file DataSheet1.docx]

**Supplementary Table S1. Ion AmpliSeq custom panel for 52 genes associated with Ehlers–Danlos syndrome and other hereditary connective tissue disorders.**

| **Disorder** | **Associated gene(s)** |
| --- | --- |
| Ehlers–Danlos syndrome | *ADAMTS2, AEBP1, B3GALT6, B4GALT7, C1R, C1S, CHST14, COL1A1, COL1A2, COL3A1, COL5A1, COL5A2, COL12A1, DSE, FKBP14, PLOD1, PRDM5, SLC39A13, TNXB, ZNF469* |
| Marfan syndrome | *FBN1* |
| Loeys–Dietz syndrome | *SMAD2, SMAD3, TGFB2, TGFB3, TGFBR1, TGFBR2* |
| Familial thoracic aortic aneurysms and aortic dissections | *ACTA2, COL3A1, FBN1, LOX, MYH11, MYLK, SMAD3, TGFB2, TGFBR1, TGFBR2* |
| Arterial tortuosity syndrome | *SLC2A10* |
| Beals syndrome | *FBN2* |
| Shprintzen–Goldberg Syndrome | *SKI* |
| FLNA-related periventricular nodular heterotopia/Otopalatodigital syndrome | *FLNA* |
| Ectopia lentis | *ADAMTSL4* |
| Osteogenesis imperfecta | *BMP1, COL1A1, COL1A2, CRTAP, FKBP10, IFITM5, LEPRE1, PLOD2, PPIB, SEC24D, SERPINF1, SERPINH1, SP7, TMEM38B, WNT1* |
| Osler disease | *ACVRL1, ENG, SMAD4* |
